# Biological and antimicrobial properties of the association Ambroxol and a water-soluble viscous liquid as a vehicle for a tricalcium silicate-based sealer

**DOI:** 10.1007/s10856-021-06604-9

**Published:** 2021-11-24

**Authors:** Índia Olinta de Azevedo Queiroz, Thiago Machado, Camila Carneiro Alves, Victor Gustavo Balera Brito, Bruno Carvalho de Vasconcelos, João Eduardo Gomes-Filho, Edilson Ervolino, Sandra Helena Penha de Oliveira, Marco Antonio Hungaro Duarte

**Affiliations:** 1 Department of Dentistry, Endodontics and Dental Materials, University of São Paulo (USP), Bauru School of Dentistry, Alameda Octávio Pinheiro Brisolla, Bauru, SP 9-75 Brazil; 2 Department of Oral and Maxillofacial Surgery and Integrated Clinic, School of Dentistry, Araçatuba, São Paulo State University (Unesp), Araçatuba, SP 1193 Brazil; 3 Department of Endodontics, School of Dentistry, Araçatuba, São Paulo State University (Unesp), Rua José Bonifácio, Araçatuba, SP 1193 Brazil; 4 Department of Basic Science, School of Dentistry, Araçatuba, São Paulo State University (Unesp), Rua José Bonifácio, Araçatuba, SP 1193 Brazil; 5 Department of Dentistry, School of Dentistry of Sobral, Federal University of Ceará (UFC), Rua Coronel Estanislau Frota, Sobral, CE 563 Brazil

## Abstract

This study aimed to investigate the antimicrobial and biological properties of Ambroxol associated with glycerin (GLI), propylene glycol (PG), and polyethylene glycol (PEG) as a possible vehicle for an experimental tricalcium silicate sealer, with the intention of developing a new biomaterial. Mouse undifferentiated dental pulp cells (OD-21) were cultured, and the effects of different association on cell proliferation and inflammatory cytokine production were investigated. Antimicrobial adhesion of *Enterococcus faecalis* to setting sealers at 2 h was evaluated. Polyethylene tubes containing experimental sealers and empty tubes were implanted into dorsal connective tissues of 12 male 3- to 4-months-old Wistar rats (250–280 g). After 7 and 30 days, the tubes were removed and processed for histological and immunohistochemical analyses. ANOVA followed by Bonferroni correction and ANOVA followed by Tukey test was used for parametric data and Kruskal–Wallis followed by Dunn for nonparametric (*p* < 0.05). Cell proliferation was dose-dependent, since all association were cytotoxic at higher concentrations; however, Ambroxol–PEG showed significantly higher cytotoxicity than other association (*p* < 0.05). In addition, irrespective of the association, no cytokine production was observed in vitro. Ambroxol–GLI reduced bacterial viability, whereas Ambroxol–PEG increased (*p* < 0.05). Histological examination showed no significant difference in the inflammatory response (*p* > 0.05) and mineralization ability in all association. Additionally, *IL-1β* and *TNF-α* were upregulated on Ambroxol–PEG in relation to Control at 07 days (*p* < 0.05). Ambroxol–GLI was the best vehicle for experimental tricalcium silicate sealer, as it promoted an increase in antimicrobial activity without altering the inflammatory response or mineralization ability.

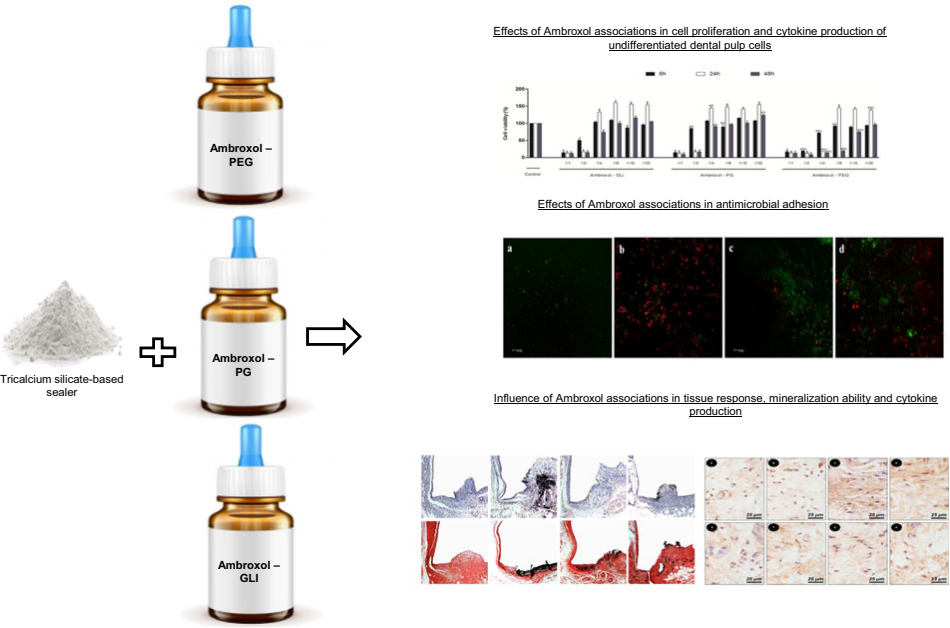

## Introduction

In the last few decades, there has been a significant improvement in the development of new biomaterials, such as tricalcium silicate-based sealers. Tricalcium silicate-based sealers are known for their satisfactory biological and physicochemical properties. Moreover, they exhibit antibacterial activity, with this property exhibited after setting, depending on the sealer’s ability to release hydroxyl ions and raise pH values [1]. These sealers are basically powder/liquid mixtures, where the powder contains the main constituents of the material, and the liquid is the vehicle for dissolving the constituents [2]. Viscous liquids, including glycerin (GLI), propylene glycol (PG), and polyethylene glycol (PEG), have been used in endodontics as vehicles owing to their adequate biological, physicochemical, and antimicrobial properties [3–5]. Moreover, it is relevant to report that setting time, ionic dissociation, tissue response, and ability to deliver materials into dentinal tubules can depend on the vehicles utilized [4, 6, 7].

The success of endodontic therapy depends on the cleaning and shaping of the root canal system, control of pathogenic microorganisms, and filling of the root canal with proper material [8, 9]. Thus, the persistence of microorganisms and their sub-products after endodontic procedures results in failure of endodontic therapy [10], especially with microorganisms that can resist modifications to environmental conditions including alkaline pH, such as *Enterococcus faecalis* [10, 11]. Antibiotics and antiseptics, both in isolation or in combination, have been employed during endodontic practice in order to improve their elimination; however, their use may cause side effects, such as development of antibiotic resistance and risk of provoked allergic reactions [12].

Ambroxol is a mucolytic expectant agent investigated for the treatment of respiratory diseases since it promotes the reduction of mucus viscosity; exhibits antioxidant, anti-inflammatory, and analgesic effects [13]; as well as shows antimicrobial properties [14]. Until now, only a few studies verifying the use of Ambroxol in dentistry have been carried out [15–17], which demonstrate that regular and prolonged use of this drug may be considered as a risk factor for dental erosion once a reduction in the mineral percentage of calcium and phosphate is detected. Even though these studies clearly demonstrate Ambroxol’s erosive effect, there is an absence of data concerning its use in the endodontic field, especially in the context of the development and/or improvement of sealers. Since success of endodontic therapy is related to the control of microorganisms, tricalcium silicate is antibacterial activity is dependent on sealer composition and the facts that the use of antibiotics and antiseptic may be promote some adverse effects. As ambroxol is a mucolytic agent that shows excellent biological properties, it is pertinent to investigate both its antimicrobial and biological effects when associated with GLI, PG, and PEG as a possible vehicle for an experimental tricalcium silicate-based sealer, with the intention of developing a new biomaterial. The null hypothesis was that there would be no difference among all vehicles in relation to both their antimicrobial and biological properties.

## Materials and methods

### Experimental groups

Before sealer preparation, 100 mg/mL of Ambroxol was added to each vehicle. The experimental sealers were mixed with each vehicle at a vehicle/powder ratio of 0.3. They were then distributed into three groups as follows: Ambroxol–GLI, Ambroxol–PG, and Ambroxol–PEG.

### In vitro study

#### Cell proliferation assay

Mouse undifferentiated dental pulp cells (OD-21) were obtained from Professor Sandra Helena Penha de Oliveira (School of Dentistry, São Paulo State University – FOA/UNESP, BR) grown under standard cell culture conditions, and cell proliferation was assessed using the 3-(4,5-dimethylthiazol-2-yl)-2,5-diphenyl tetrazolium bromide assay. Fresh sealer extracts were prepared following the method of Pedano et al. [18] and ISO 10993-12:2012 standard recommendations [19]. Serial extract dilutions (undiluted, 1/2, 3/4, 1/8,1/16,1/32) were used. Briefly, OD-21 cells were seeded into 24-well plates (1.0 × 10^5^cells/mL of medium per well), incubated at 37 °C in a humidified air atmosphere of 5% CO_2_ for 24 h, and then exposed to extracts for 6, 24 and 48 h. Control cells were cultured in media without extracts. The optical density was measured using a spectrophotometer (Spectra Max 190; Molecular Devices, CA, USA) at 570 nm wavelength.

#### Inflammatory cytokine production assay

OD-21 cells were seeded into 24-well plates (1 × 10^5^cells/well), incubated for 24 h, after which fresh extracts (1/8) were added to cells. After 6, 24, and 48 h, culture media were collected, and the levels of interleukin (IL)-1β, IL-6, tumor necrosis factor (TNF)-α, and IL-17 proteins were evaluated using DuoSet enzyme-linked immunosorbent assay kits (R&D Systems, MN, USA). Cells cultured without treatment served as controls.

#### Antimicrobial adhesion assay

Sealer discs (5 mm in diameter and 2 mm in height) were prepared and kept in an incubator at 37 °C for 72 h. They were then sterilized in ultraviolet light for 1 h. Antimicrobial adhesion was performed as described by Colombo et al. [20], with some modifications. Briefly, an inoculum of ~1 × 10^7^ (colony forming units/mL) of *Enterococcus faecalis* (ATCC 29212) was seeded onto each sealer disc and incubated at 37 °C for 2 h. The discs were then washed in phosphate-buffered saline and stained with LIVE/DEAD BacLight Bacterial Viability Kit (Invitrogen Molecular Probes, OR, USA) for 10 min. The discs were examined with a confocal laser scanning microscope (Leica TCS-SPE; Leica Microsystems GmbH, Mannheim, Germany) and analyzed using bioImage L software, which generated the percentages and biovolume of live and dead bacteria based on green and red fluorescence, respectively. In the control group, the bacteria were grown on glass coverslips.

### In vivo study

#### Subcutaneous implantation

This study was approved by the Ethical Committee (process number 01007-2018), and subcutaneous implantation was performed [21]. Polyethylene tubes (Abbott Labs of Brazil, SP, Brazil) with a 1.0 mm internal diameter, 1.6 mm external diameter, and 10.0 mm length were filled with experimental sealers. In addition, empty tubes were implanted in the connective tissue of 12 other *Wistar* rats. After 7 and 30 days of implantation, six animals from each group were euthanized with an overdose of anesthetic (100 mg/kg of Thiopentax; Cristália, São Paulo, Brazil), and the implanted tubes with the surrounding tissues were removed and processed.

#### Histological and immunohistochemical analyses

For histological analyses, the tissues were sliced into 5 μm sections and stained with hematoxylin-eosin. The 10 μm sections were stained using the von Kossa technique. The average number of cells in each group was determined from 10 separate areas (400×). Analyses were performed by a single calibrated operator in a blinded manner using light microscopy (DM 4000 B; Leica Microsystems, Wetzlar, Germany). Inflammatory reactions in the tissue that was in contact with the material on the open end of the tube were scored as described in previous studies [21], and evaluated according to ISO/TR 7405-1997 as: score 0, no or few inflammatory cells and no reaction; score 1, <25 cells and a mild reaction; score 2, 25–125 cells and a moderate reaction; and score 3, ≥125 cells and a severe reaction.

For immunohistochemical analyses, the tissues were sliced into 5μm semi-serial sections and subjected to immunohistochemistry to detect IL-1β (Rabbit anti-IL-1β orb 101745), TNF-α (Rabbit TNF-α orb 11495), IL–6 (Rabbit anti-IL-6 orb 6210), and IL–17 (rabbit anti-IL-17 SC 7927). A certified histologist (EE), who was blinded to the treatments, performed the analyses. The criteria for immunoreactivity were as follows: score 0: total absence of immunoreactivity (IR); score 1 (low): IR in ~25% of cells per field; score 2 (moderate): IR in ~50% of cells per field; and score 3 (high): IR in ~75% of cells per field [21].

### Statistical analysis

Graph Pad Prism (version 9.0) software program was used for statistical analysis. The *p* value was considered significant at 5%. All data were subjected to the D’Agostino & Pearson normality test. Data regarding cell viability and cytokine production were analyzed statistically by ANOVA followed by Bonferroni correction. Data regarding antimicrobial adhesion were analyzed by ANOVA followed by a Tukey test. A Kruskal–Wallis test followed by Dunn’s test was used for histological and immunohistochemical analyses.

## Results

### Effects of Ambroxol associations on cell proliferation and cytokine production in undifferentiated dental pulp cells

**Fig. 1:**
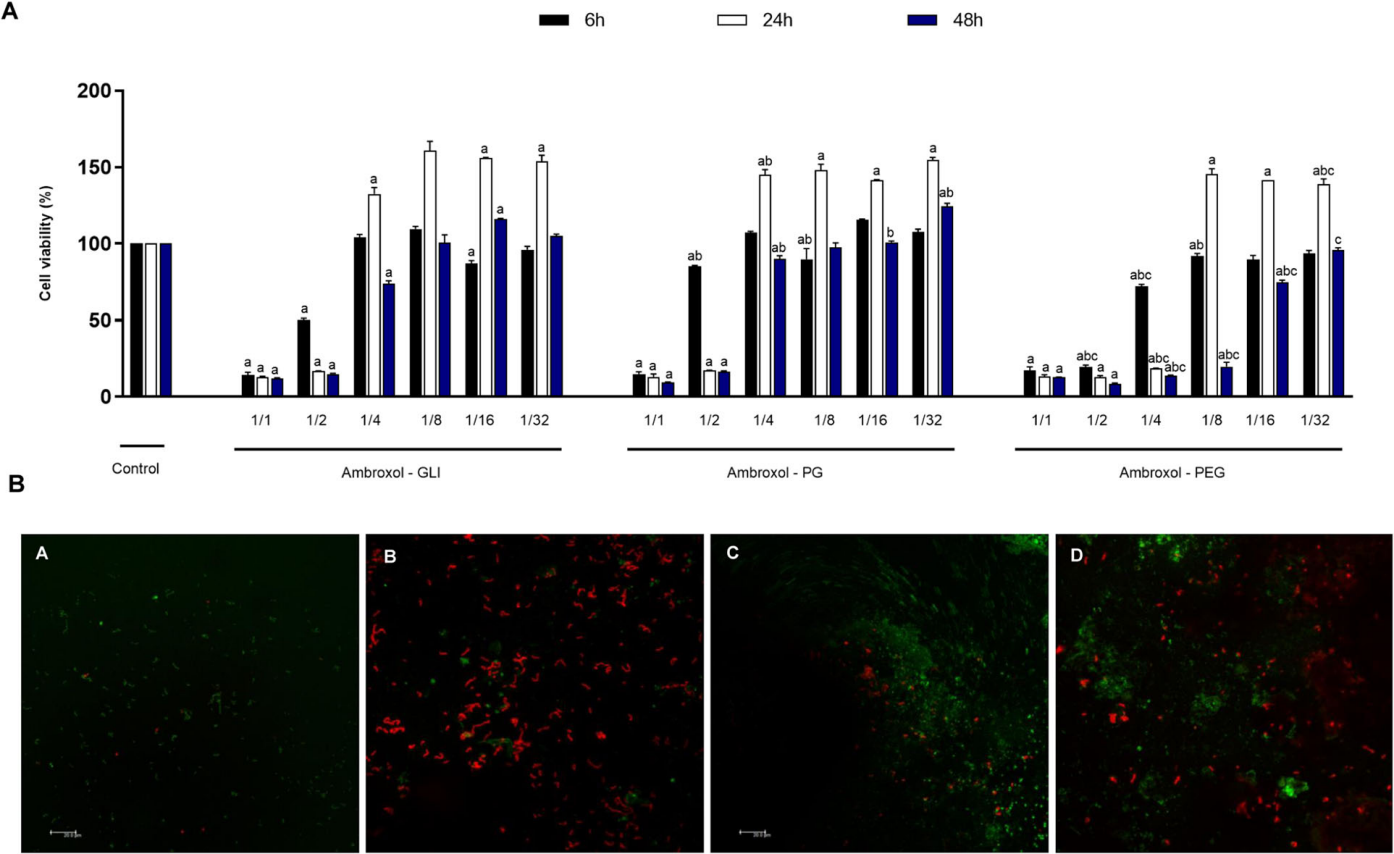
**A** Cell viability observed after stimulation with diluted extracts of the Ambroxol associations at 6, 24 and 48 h. The letters indicate statistical difference when comparing different association at the same dilution. (a) *p* < 0.05 versus Control; (b): *p* < 0.05 versus Glycerin; (c): *p* < 0.05 versus Propylene Glycol. **B** Confocal laser scanning microscopy images of antimicrobial adhesion to sealer after setting after 2 h: (a) Control (coverslip); (b) Ambroxol-associated glycerin; (c) Ambroxol-associated Propylene Glycol; (d) Ambroxol-associated Polyethylene Glycol 400. Green and red fluorescence represents live and death microorganisms, respectively. Scale bars: 20 µm

Cell proliferation was dose-dependent, as exposure to lower dilutions (undiluted, ½, and ¼) promoted a reduction in cell metabolism when compared with the control group at all times evaluated (*p* < 0.05), except for ¼ dilution of Ambroxol–GLI and Ambroxol–PG, which exhibited increased cell growth at 24 h of 32 and 44%, respectively (*p* < 0.05). Furthermore, exposure to higher dilutions (1/8, 1/16, 1/32) of Ambroxol–GLI stimulated a raise in proliferation rate of 60; 56; 53%, respectively; Ambroxol–PG of 48; 41; 54%, respectively; and Ambroxol–PEG of 45; 41; 38%, respectively at 24 h (*p* < 0.05); however, there was a decrease at 48 h in the Ambroxol–PEG 1/8 dilution (81%) (*p* < 0.05) (Fig. 1).

Comparison between extracts at the same dilution showed diminished cell metabolism in Ambroxol–PEG (½ and ¼) compared to Ambroxol–GLI at 6 h of 31 and 32%, respectively; at 24 h of 4 and 114%, respectively; and at 48 h of 6 and 50%, respectively; a reduction was also observed when compared with Ambroxol–PG at 6 h (66 and 35%); 24 h (5 and 126%) and 48 h (8 and 77%) (*p* < 0.05). In addition, a decrease was observed in the presence of Ambroxol–PEG 1/8 and 1/16 dilution at 48 h compared with Ambroxol–GLI (81 and 88%, respectively) and Ambroxol–PG (41 and 96%, respectively; *p* < 0.05). On the other hand, it was found that between Ambroxol–GLI and Ambroxol–PG, 1/8 dilution of Ambroxol–GLI stimulated cell growth at 6 h of 11% (*p* < 0.05), whereas an increase at 24 h of 12%, and at 48 h, 17% of proliferation was identified in Ambroxol–PG (*p* < 0.05) (Fig. 1).

Irrespective of the association evaluated, no cytokine production was observed.

### Effects of Ambroxol associations on antimicrobial adhesion

A similar initial biovolume was observed (*p* > 0.05). After 2 h, Ambroxol–PEG exhibited the highest percentage of viable bacteria in contrast to Ambroxol–GLI, which showed the lowest values when compared to the other and the control group (*p* < 0.05) (Fig. 1).

### Influence of Ambroxol associations on tissue response, mineralization ability, and cytokine production

**Fig. 2:**
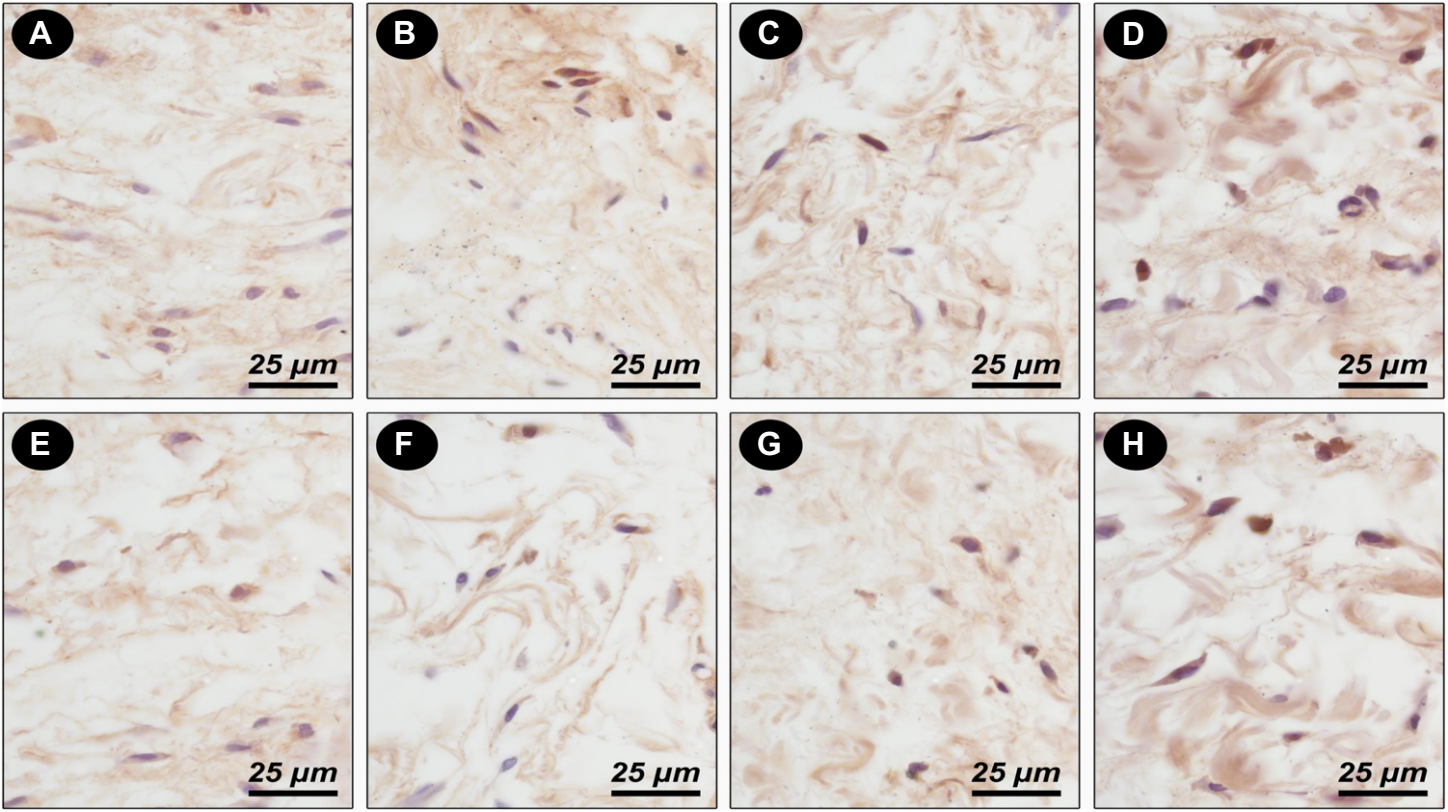
Photomicrographs showing the immunoreactive cells (arrowheads) for *TNF-α* (**A**–**D**); *IL-1β* (**E**–**H**) at 30 days for control (**A**, **E**); Ambroxol-associated Propylene Glycol (**B**, **F**); Ambroxol-associated glycerin (**C**, **G**); Ambroxol-associated Polyethylene Glycol 400 (**D**, **H**). Harris-hematoxylin counterstaining. Scale bars: 25 μm

**Fig. 3:**
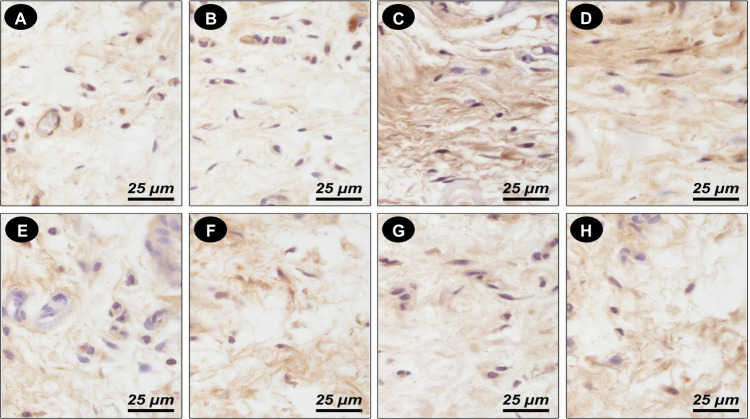
Photomicrographs showing the immunoreactive cells (arrowheads) for *IL-6* (**A**–**D**); *IL-17* (**E**–**H**) at 30 days for Control (**A**, **E**); Ambroxol-associated Propylene Glycol (**B**, **F**); Ambroxol-associated glycerin (**C**, **G**); Ambroxol-associated Polyethylene Glycol 400 (**D**, **H**). Harris-hematoxylin counterstaining. Scale bars: 25 μm

In the control group, mild inflammatory cell infiltration consisting of macrophages and lymphocytes was observed in the thin fibrous capsule. The empty tubes were not positive for von Kossa staining. On the other hand, moderate inflammatory cell infiltration consisting of macrophages and lymphocytes, as well as a thick fibrous capsule formation, was observed on day 7, and a mild inflammatory response with a thin fibrous capsule was identified on day 30 in all associations investigated. In addition, von Kossa positivity was observed in all groups at days 7 and 30 (*p* < 0.05) (Table 1). In addition, IL-1β, TNF-α, IL–6, and IL–17–positive cells were present in all associations evaluated at 7 and 30 days. Moreover, the Ambroxol–PEG group produced more IL-1β and TNF-α than the control group at day 7 (*p* < 0.05) (Figs. 2 and 3).

**Table 1:** Inflammatory scores specimens stained with hematoxylin-eosin, thickness of fibrous capsule and presence of mineralization in all groups

Time	Material	Inflammatory response				Capsule	Mineralization
		Scores					Von Kossa
		0	1	2	3		
7 days	Control	0/6	4/6	2/6	0/6	Thin	Absent
	Ambroxol-GLI	0/6	2/6	3/6	1/6	Thick	Present
	Ambroxol-PG	0/6	0/6	6/6	0/6	Thick	Present
	Ambroxol-PEG	0/6	0/6	5/6	1/6	Thick	Present
30 days	Control	0/6	5/6	1/6	0/6	Thin	Absent
	Ambroxol-GLI	0/6	4/6	2/6	0/6	Thin	Present
	Ambroxol-PG	0/6	4/6	2/6	0/6	Thin	Present
	Ambroxol-PEG	0/6	4/6	0/6	0/6	Thin	Present

*GLI* glycerin, *PG* propylene glycol, *PEG* polyethylene glycol 400

## Discussion

Several investigations have been carried out to identify the most appropriate substance (liquid or powder) that can be added to endodontic sealers without interfering with its properties [3, 4]. Therefore, the main purpose of this study was to develop a new material using Ambroxol because of its antioxidant, anti-inflammatory, analgesic, and antimicrobial properties. Toward this, a small amount of Ambroxol was associated with different vehicles aimed at improving the antimicrobial and biological properties of this experimental endodontic sealer. The null hypothesis was rejected since Ambroxol–GLI was found to be the best vehicle for the experimental tricalcium silicate sealer, which this association promoted an increase in antimicrobial activity without affecting the inflammatory response or mineralization ability of teeth. To the best of our knowledge, this is the first study to assess the use of ambroxol in the endodontic field, especially in the context of the development of new biomaterials.

In general, cells treated with lower dilutions of Ambroxol associations exhibited lower cell proliferation, indicating cytotoxicity. Conversely, cells subjected to higher dilutions showed increased proliferation at 24 h. Yamaya et al. [22] demonstrated that treatment of human tracheal epithelial cells with Ambroxol did not change cell viability. Our results agree with those of Sunkari et al. [23], which showed that the effect of Ambroxol was dose-dependent, since no significant reduction in murine macrophage viability up to 2.5 μM was detected, whereas in keratinocytes, less cytotoxicity was found up to 5 μM. Our data also revealed that Ambroxol–PEG impaired the proliferation rate compared with other associations. No difference in cytotoxicity in rabbit corneal epithelial cells among the vehicles was reported by Onizuka et al. [24]. In contrast, PEG created a stressful environment for cells (intracellular hypoxia), which led to alterations in energy metabolism [25]. Therefore, we believe that these other findings can explain our results.

Although studies have shown that GLI, PG, and PEG induce the release of inflammatory cytokines [26, 27], no cytokine synthesis has been seen irrespective of their association in vitro. Gibbs et al. [28] reported that Ambroxol inhibited the release of histamine, leukotrienes, and cytokines from human leukocytes and mast cells. Yamaya et al. [22] showed that Ambroxol reduced cytokine production in the supernatant of human tracheal epithelial cells infected with rhinovirus. Moreover, cytokine secretion has been related to the concentration of Ambroxol administered [29]. Cytokine production depends on the cell type, sealer composition, and cytokines investigated [21, 23, 29]. Therefore, we believe that the undifferentiated cell line and the concentration of Ambroxol utilized may be the reason for this absence, and could therefore justify our findings. However, further investigation is required to elucidate these mechanisms.

Endodontic sealers should exhibit antimicrobial activity even after setting to avoid bacterial adhesion and biofilm formation on the tooth’s surface. Based on this issue, an antimicrobial adhesion assay was performed that showed that Ambroxol–GLI reduced *E. faecalis* viability, whereas Ambroxol–PEG increased. Safavi and Nakayama [6] reported that high concentrations of GLI or pure PG reduced the conductivity of calcium hydroxide, which may affect their dissociation and decrease their effectiveness. In contrast, alkaline pH and calcium release in the presence of GLI have been reported [30, 31]. Our results are supported by the findings of Gomes et al. [5], who demonstrated that GLI produced larger zones of microbial growth inhibition, whereas PEG showed the weakest antimicrobial action. Additionally, a reduction in cell adherence by Ambroxol was reported by Kong et al. [14]. Furthermore, it is important to highlight that the type of vehicle affects diffusion and antimicrobial activity because viscous vehicles release Ca^+2^ and OH^−^ ions more slowly [5]. Thus, the type and characteristics of materials, such as their superficial microstructure, surface roughness, and fissures, can influence bacterial adhesion and biofilm formation [20].

Despite the differences between our in vitro and in vivo results, it is essential to emphasize that cell culture studies did not characterize the underlying host-response interaction. In the in vivo study, irrespective of the association, the moderate inflammatory response decreased with time, immunoreactive cells were identified, and IL-1β and TNF-α production were upregulated by Ambroxol–PEG compared to the control at 7 days. However, researchers have reported the toxic effects of vehicles [32, 33], with our findings confirmed by early reports [4]. In addition, the release of pro-inflammatory cytokines after exposure to both vehicle and Ambroxol has been previously described [23, 32]. IL-1β and TNF-α are cytokines involved in several of cellular events, although other proinflammatory mediators are also responsible for perpetuating the inflammatory response [34]. The toxic and irritant effects of PEG have been widely reported [32, 35]. The ionic dissociation promoted by PEG leads to delayed release of Ca^+2^ e OH^−^ ions [36]; thus, the increase in Ca^+2^ ions may act as a tissue irritant, and, at the same time, the alkaline environment provided by OH^−^ ions, which is associated with the ability of experimental sealers to precipitate apatite during the acute phase of inflammation [37], can stimulate the release of these specific cytokines. Taken together, these facts may explain our data. Nevertheless, further investigation is required to clarify these findings.

Deposition of mineralized tissue is associated with the ability of biomaterials to release Ca^+2^ ions [21]. Studies have shown that Ambroxol blocks calcium and sodium channels [38] and reduces the mineral percentage of calcium and phosphate in dentin [15–17]. In this study, mineralization areas were identified, showing that any experimental sealer inhibited calcium release. Therefore, it is possible that the concentration of Ambroxol used was not able to alter and/or interfere with both calcium release and calcium homeostasis, which may justify our findings. Moreover, calcium released in the presence of GLI, PG, and PEG has already been described [4, 30].

## Conclusion

Our results showed that the Ambroxol–GLI association was the best vehicle among those used, because it promoted an increase in antimicrobial activity without altering the inflammatory response or mineralization ability of teeth, whereas the Ambroxol–PEG association negatively interfered with cell proliferation, reduced antimicrobial activity, and created an inflammatory microenvironment.
